# Association between Information and Communication Technology use and Ocular Axial Length Elongation among Middle-Aged Male Workers

**DOI:** 10.1038/s41598-019-53423-8

**Published:** 2019-11-25

**Authors:** Toru Honda, Toru Nakagawa, Yuya Watanabe, Takeshi Hayashi, Tadashi Nakano, Seichi Horie, Masayuki Tatemichi

**Affiliations:** 1 Hitachi Health Care Center, Ibaraki, Japan; 2 Department of Ophthalmology, The Jikei University School of Medicine, Tokyo, Japan; 3 Department of Health Policy and Management, University of Occupational and Environmental Health, Kitakyushu, Japan; 4 Department of Preventive Medicine, Tokai University, School of Medicine, Kanagawa, Japan

**Keywords:** Occupational health, Risk factors

## Abstract

The use of Information and Communication Technology devices (ICT) has spread globally; therefore, increasing exposure to these display devices is an important health issue in the workplace. The association between ocular axial length (AL) elongation and ICT use was investigated among 7334 middle-aged Japanese male workers. Detailed ICT use information was obtained using a self-administered questionnaire. The high AL group was defined as the group with AL above the 75th percentile for each 5-year age interval. Logistic analysis showed that the odds ratio (OR) for the high AL group of >8 hours/day, adjusted for potent confounding factors, was significantly elevated relative to <1 hour/day. Notably, there was a strong association in the older groups (OR = 6.60, 95% CI = 3.92–11.12), based on work-related uses: word processing, sending e-mails, preparation of presentation materials, and browsing websites. In addition, among workers with extended ICT use for these work-related purposes, a significant lateral difference was observed in AL. However, these associations were not observed for private use, such as playing games. The results of our prospective cross-sectional study show that increased time spent on work-related ICT uses was associated with AL elongation, particularly in older workers.

## Introduction

Over the past few decades, the duration of use of display devices with backlight illumination, such as computer monitors, smartphones, and tablets (collectively referred to as Information and Communication Technology [ICT] devices) has been increasing in occupational and private activities^1^. Notably, increasing exposure to these display devices is an important health issue in the workplace, which has been linked to visual display terminal syndrome^2,3^. ICT eye diseases, including asthenopia and dry eye, are universal concerns^1^.

In recent years, the prevalence of myopia, particularly axial myopia in which the ocular axial length (AL) is stretched in the long axis direction, has been increasing globally, such that it comprises an important public health problem in both developing and developed countries^4,5^. Many reports show that education^6^, time spent outdoors^7^, and physical activity^8^ are important causal environmental factors associated with myopia risk. In addition, a recent systematic review has shown that near work activity is associated with myopia^9^. Although there are no obvious indications that the increasing prevalence of myopia is related to the use of ICT, increased near work (e.g., use of ICT during periods of growth) may cause myopia by elongating the AL^1,9^.

In addition to reductions in visual ability from myopia, the risk of glaucoma is increased by 1.50-fold to 4.67-fold^10–18^. However, the detailed pathological mechanisms underlying the effect of axial myopia in the development of glaucoma remain largely unknown. It has been reported that disturbances in the blood supply to the eye, caused by overextension of the retina due to AL prolongation, contribute to the progress of glaucoma^19–21^.

We have previously reported a relationship between ICT use and glaucomatous visual field abnormalities in cross-sectional and cohort study designs^22–24^. As an underlying mechanism, we hypothesised that an association might exist between ICT use and elongation of the AL in adults. Therefore, we collected large-scale data relating to AL to test our hypothesis in this cross-sectional study; after adjusting for known confounding factors, we sought to clarify the association between ICT use and elongation of AL.

## Results

Table 1 shows the participants’ background characteristics and ICT use. There were significant differences in age, height, history of glaucoma, and history of ocular hypertension among the five categories of ICT use. Particularly, the <1 hour of ICT use group was significantly older than the >12 hours of ICT use group (mean 53.4 ± 8.4 years *vs*. 46.2 ± 6.5 years; one-way ANOVA). Height increased with ICT use (169.6 ± 6.3 cm in the <1 hour of ICT use group, compared with 172.1 ± 6.1 cm in the >12 hours of ICT use group). There were significant differences among the five groups in history of glaucoma and ocular hypertension, but no clear tendencies were present (Table 1). AL was significantly longer in the >12 hours of ICT use group (25.66 ± 1.47 mm) compared with the <1 hour of use group (24.54 ± 1.35 mm; one-way ANOVA) (p < 0.001); a clear tendency was observed (Table 1).

**Table 1:** Characteristics of Participants.

		ICT use time per day						Total
		none or <1 hr.	1–4 hr.	4–8 hr.	8–12 hr.	≧12 hr.	*p*	
Number		1136	1996	1970	1789	443		7334
Age (years old)	Mean	53.4	48.7	51.7	49.3	46.2	<0.001*	50.2
	SD	8.4	8.3	7.4	7.0	6.5		7.9
Height (cm)	Mean	169.6	171.1	171.3	171.8	172.1	<0.001*	171.2
	SD	6.3	6.1	5.9	5.8	6.1		6.1
Glaucoma	number	47	60	98	90	19	0.013**	314
	%	4.1%	3.0%	5.0%	5.0%	4.3%		4.3%
History of ocular hypertension	number	95	118	176	137	40	0.005**	566
	%	8.4%	5.9%	8.9%	7.7%	9.0%		7.7%
Family history of glaucoma	number	98	176	198	178	52	0.222**	702
	%	8.6%	8.8%	10.1%	9.9%	11.7%		9.6%
Axial length (right) (mm)	Mean	24.54	24.79	25.27	25.52	25.66	<0.001*	25.11
	SD	1.35	1.32	1.47	1.49	1.47		1.47
Axial length (left) (mm)	Mean	24.50	24.74	25.21	25.44	25.58	<0.001*	25.05
	SD	1.35	1.29	1.45	1.47	1.45		1.45

*Statistical significance was determined by one-way ANOVA.

**Statistical significance was determined by Chi-squired test.

SD = standard deviation.

There were no significant differences between left and right sides within 5-year age intervals (Supplemental Table 1). When comparing between 5-year age intervals, the 35–40 years group showed ALs of 25.29 and 25.24 mm on the right and left, respectively. Conversely, the 61–65 years group showed ALs of 24.74 and 24.67 mm on the right and left, respectively. This indicated that younger participants tended to have ALs that were significantly longer in both left and right eyes (Supplemental Table 1).

**Figure 1:**
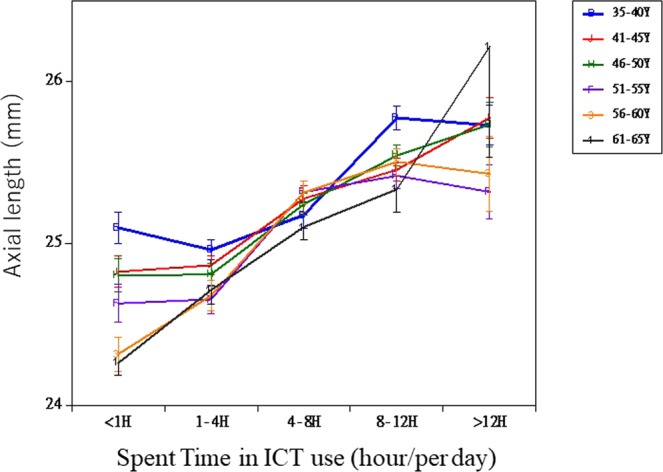
Association between axial length and Information and Communication Technology device use, stratified by age. Axial length was adjusted for height by using ANCOVA. Data are presented as mean ± standard error of the mean.

The respective values of the 75th percentile high AL were 26.20, 25.99, 26.11, 26.11, 26.12, and 25.63, in the 5-year age interval groups of 35–40, 41–45, 46–50, 51–55, 56–60, and 61–65, respectively. When ICT use was >4 hours/day, there was a significant association with high AL among all participants aged ≥41 years. In particular, the OR tended to increase in the groups with older age (Table 2 and Fig. 1). This relationship did not differ between models 1 and 2.

**Table 2:** Association between total ICT use time and axial length elongation by stratification with age.

Age	ICT Use Time/day	n	model 1				model 2			
			Odds	95% CI		p	Odds	95% CI		p
35–40 y	none or <1 hr.	67	Reference				Reference			
	1–4 hr.	331	0.69	0.36	1.32	0.261	0.67	0.35	1.28	0.229
	4–8 hr.	147	0.90	0.44	1.81	0.760	0.87	0.43	1.75	0.690
	≧8 hr	289	1.76	0.94	3.30	0.077	1.75	0.93	3.28	0.083
			*p* for trend < 0.001				*p* for trend < 0.001			
41–45 y	none or <1 hr.	201	Reference				Reference			
	1–4 hr.	513	1.35	0.84	2.15	0.216	1.33	0.83	2.14	0.233
	4–8 hr.	286	2.30	1.42	3.74	0.001	2.27	1.39	3.70	0.001
	≧8 hr	498	3.77	2.41	5.89	<0.001	3.78	2.41	5.92	<0.001
			*p* for trend < 0.001				*p* for trend < 0.001			
46–50 y	none or <1 hr.	192	Reference				Reference			
	1–4 hr.	395	0.80	0.50	1.27	0.347	0.80	0.50	1.27	0.339
	4–8 hr.	427	1.65	1.08	2.54	0.022	1.61	1.05	2.48	0.030
	≧8 hr	553	2.20	1.46	3.32	<0.001	2.13	1.41	3.23	<0.001
			*p* for trend < 0.001				*p* for trend < 0.001			
51–55 y	none or <1 hr.	163	Reference				Reference			
	1–4 hr.	284	0.95	0.54	1.68	0.865	0.98	0.55	1.73	0.941
	4–8 hr.	445	2.53	1.54	4.15	<0.001	2.58	1.56	4.25	<0.001
	≧8 hr	464	2.74	1.67	4.48	<0.001	2.79	1.70	4.60	<0.001
			*p* for trend < 0.001				*p* for trend < 0.001			
56–60 y	none or <1 hr.	178	Reference				Reference			
	1–4 hr.	217	1.63	0.84	3.14	0.148	1.66	0.86	3.23	0.132
	4–8 hr.	384	4.48	2.52	7.94	<0.001	4.31	2.41	7.70	<0.001
	≧8 hr	328	5.53	3.11	9.85	<0.001	5.39	3.01	9.66	<0.001
			*p* for trend < 0.001				*p* for trend < 0.001			
61–65 y	none or <1 hr.	335	Reference				Reference			
	1–4 hr.	256	1.94	1.23	3.05	0.004	1.98	1.26	3.12	0.003
	4–8 hr.	281	4.12	2.72	6.26	<0.001	4.16	2.72	6.35	<0.001
	≧8 hr	100	6.60	3.92	11.12	<0.001	6.75	3.98	11.43	<0.001
			*p* for trend < 0.001				*p* for trend < 0.001			

The high AL group was determined as above the 75th percentile for each 5-year age interval. The odds ratio for the high AL group was calculated using the logistic model.

Model 1: Abjuseted for age and height.

Model 2: Adjusted for age, height, exercise, and history of ocular hypertension.

95%CI = 95% confidence interval.

Next, we investigated whether the type of ICT used for work was associated with AL elongation. The use of word processing and email applications for a duration of more than one hour was significantly associated with ocular AL elongation in all age groups. This association was also seen in most age groups when the use of ICT included the assembly of presentation materials (Table 3 and Supplemental Table 2). However, the associations with email and web browsing tasks were not particularly robust in private use among any of the age groups (Table 4). In addition, there were no significant associations with the spent time playing games in any of the age groups (Table 4). Additional adjustments for physical activity and history of ocular hypertension did not affect these associations (data not shown).

**Table 3:** Association between work related ICT use time and axial length elongation by stratification with age.

Age	ICT Use Time/day	Word processing					Assembling presentation materials					Sending e-mails				
		n	Odds	95% CI		p	n	Odds	95% CI		*p*	n	Odds	95% CI		*p*
35–40 y	none or <1 hr.	289	Reference				484	Reference				268	Reference			
	1–4 hr.	344	2.52	1.69	3.77	<0.001	294	1.62	1.16	2.26	0.004	486	2.93	1.95	4.42	<0.001
	≧4 hr.	196	2.57	1.64	4.02	<0.001	51	1.59	0.83	3.02	0.160	75	3.84	2.12	6.97	<0.001
			*p* for trend < 0.001					*p* for trend=0.007					*p* for trend < 0.007			
41–45 y	none or <1 hr.	467	Reference				937	Reference				446	Reference			
	1–4 hr.	652	2.63	1.90	3.65	<0.001	471	2.05	1.59	2.63	<0.001	881	3.36	2.42	4.67	<0.001
	≧4 hr.	374	3.94	2.78	5.59	<0.001	85	2.24	1.39	3.60	0.001	166	4.03	2.60	6.24	<0.001
			*p* for trend < 0.001					*p* for trend<0.001					*p* for trend < 0.001			
46–50 y	none or <1 hr.	380	Reference				855	Reference				336	Reference			
	1–4 hr.	758	2.31	1.65	3.21	<0.001	605	1.83	1.43	2.32	<0.001	1024	2.42	1.71	3.40	<0.001
	≧4 hr.	419	2.85	1.99	4.06	<0.001	97	1.70	1.07	2.72	0.026	197	3.46	2.25	5.32	<0.001
			*p* for trend < 0.001					*p* for trend< 0.001					*p* for trend < 0.001			
51–55 y	none or <1 hr.	248	Reference				727	Reference				217	Reference			
	1–4 hr.	679	2.98	1.94	4.57	<0.001	533	1.87	1.44	2.43	<0.001	945	2.67	1.73	4.13	<0.001
	≧4 hr.	417	3.16	2.02	4.95	<0.001	84	1.94	1.18	3.20	0.009	182	3.35	2.00	5.62	<0.001
			*p* for trend < 0.001					*p* for trend< 0.001					*p* for trend < 0.001			
56–60 y	none or <1 hr.	224	Reference				590	Reference				190	Reference			
	1–4 hr.	592	3.10	1.97	4.88	<0.001	435	1.72	1.29	2.29	<0.001	752	3.40	2.06	5.61	<0.001
	≧4 hr.	272	4.32	2.66	7.02	<0.001	63	1.64	0.92	2.91	0.094	146	6.51	3.65	11.60	<0.001
			*p* for trend < 0.001					*p* for trend = 0.001					*p* for trend < 0.001			
61–65 y	none or <1 hr.	258	Reference				542	Reference				241	Reference			
	1–4 hr.	369	1.92	1.37	2.69	<0.001	191	1.53	1.07	2.18	0.020	477	2.42	1.75	3.35	<0.001
	≧4 hr.	134	3.33	2.17	5.11	<0.001	28	3.83	1.78	8.25	0.001	43	3.47	1.78	6.78	<0.001
			*p* for trend < 0.001					*p* for trend< 0.001					*p* for trend < 0.001			

The high AL group was determined as above the 75th percentile for each 5-year age interval. The odds ratio for the high AL group was calculated using the logistic model after adjusted for age and height. 95%CI = 95% confidence interval.

**Table 4:** Association between private use time of ICT or playing games and axial length elongation by stratification with age.

Age	ICT Use Time/day	Private Use					ICT Use Time/day	Playing games				
		n	Odds	95% CI		*p*		n	Odds	95% CI		*p*
35–40 y	none or <1 hr.	132	Reference									
	1–4 hr.	555	1.24	0.78	1.97	0.363	none or <1 hr.	336	Reference			
	4–8 hr.	87	1.23	0.65	2.34	0.528	1–4 hr.	471	0.97	0.70	1.34	0.837
	≧8 hr.	60	1.53	0.76	3.09	0.235	≧4 hr.	27	0.67	0.24	1.83	0.431
			*p* for trend = 0.275						*p* for trend = 0.661			
41–45 y	none or <1 hr.	376	Reference									
	1–4 hr.	883	1.19	0.89	1.59	0.232	none or <1 hr.	781	Reference			
	4–8 hr.	139	0.96	0.60	1.54	0.862	1–4 hr.	683	0.93	0.73	1.18	0.546
	≧8 hr.	100	1.86	1.15	3.01	0.012	≧4 hr.	34	0.57	0.23	1.42	0.229
			*p* for trend = 0.060						*p* for trend = 0.356			
46–50 y	none or <1 hr.	512	Reference									
	1–4 hr.	780	0.97	0.75	1.26	0.840	none or <1 hr.	972	Reference			
	4–8 hr.	173	1.12	0.75	1.66	0.581	1–4 hr.	568	0.88	0.69	1.12	0.294
	≧8 hr.	102	1.17	0.72	1.88	0.531	≧4 hr.	27	0.66	0.25	1.76	0.404
			*p* for trend = 0.485						*p* for trend = 0.221			
51–55 y	>1 h	478	Reference									
	1–4 hr.	619	1.07	0.81	1.42	0.643	none or <1 hr.	935	Reference			
	4–8 hr.	165	0.87	0.56	1.34	0.523	1–4 hr.	407	0.80	0.60	1.05	0.108
	≧8 hr.	94	2.01	1.26	3.23	0.004	≧4 hr.	14	0.00	0.00	-	0.999
			*p* for trend = 0.069						*p* for trend = 0.037			
56–60 y	none or <1 hr.	474	Reference									
	1–4 hr.	425	0.87	0.64	1.20	0.403	none or <1 hr.	854	Reference			
	4–8 hr.	155	1.17	0.77	1.76	0.457	1–4 hr.	248	1.12	0.81	1.55	0.487
	≧8 hr.	53	1.68	0.92	3.08	0.092	≧4 hr.	5	4.79	0.79	29.00	0.088
			*p* for trend = 0.182						*p* for trend = 0.297			
61–65 y	none or <1 hr.	530	Reference									
	1–4 hr.	313	1.42	1.03	1.97	0.035	none or <1 hr.	819	Reference			
	4–8 hr.	106	2.14	1.36	3.37	0.001	1–4 hr.	147	1.21	0.82	1.80	0.338
	≧8 hr.	23	1.91	0.78	4.65	0.154	≧4 hr.	6	1.64	0.30	9.05	0.572
			*p* for trend < 0.001						*p* for trend = 0.288			

The high AL group was determined as above the 75th percentile for each 5-year age interval. The odds ratio for the high AL group was calculated using the logistic model after adjusted for age and height. 95%CI = 95% confidence interval

95%CI = 95% confidence interval.

**Figure 2:**
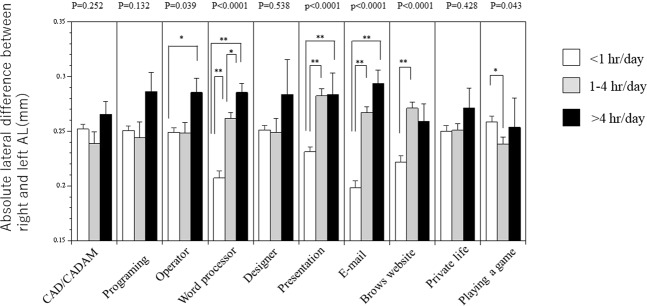
Absolute lateral difference between right and left axial length by Information and Communication Technology device (ICT) use. There were significant differences in ICT use per day for word processing, assembly of presentation materials, email, and website browsing. Data are presented as mean ± standard error. Significance was determined by one-way ANOVA and Bonferroni correction. *P < 0.05, **P < 0.01.

We examined differences in AL between right and left eyes. Interestingly, there were significant differences in ICT use among various tasks (i.e., word processing, presentation, preparation of e-mail, sending e-mail, and browsing websites; Fig. 2).

We investigated the associations between glaucoma and AL. In the present study, 314 workers had been diagnosed with glaucoma (mean age, 53.5 ± 7.3 years). The risk of glaucoma was significantly associated with age, history of ocular hypertension (OR = 18.6, 95% CI = 14.37–24.10), family history of glaucoma (OR = 2.17, 95% CI = 1.56–3.02), and AL longer than 75th percentile (OR = 5.53, 95% CI = 3.71–8.23; Table 5).

**Table 5:** Axial length as a risk for glaucoma.

		n	Odds	95% CI		p
Age		7334	1.04	1.03	1.06	0.000
Hight		7334	0.99	0.97	1.01	0.352
History of ocular hypertension	non	566		Reference		
	present	6768	18.61	14.37	24.10	<0.001
Family history of Glaucoma	non	702		Reference		
	present	6632	2.17	1.56	3.02	<0.0001
Axial length	<25% tile	1846		Reference		
	25–50% tile	1832	1.22	0.74	2.00	0.443
	50–75% tile	1833	2.04	1.31	3.17	0.002
	>75% tile	1823	5.53	3.71	8.23	<0.001
				*p* for trend < 0.001		

Odds ratio for subjects who had a history of glaucoma was calculated using logistic model.

95%CI = 95% confidence interval.

## Discussion

This study demonstrated that increased ICT use was significantly associated with longer AL among middle-aged Japanese male workers. Furthermore, in older age groups, the extent of ICT use tended to show a greater influence on AL. In addition, high AL was strongly related to ICT use for work, but not to ICT use for personal tasks (i.e., playing a game). These findings are novel, and to the best of our knowledge, we believe that this is the first report regarding ICT use and AL.

In this study, ocular AL was found to be significantly longer in younger participants, even after adjustment for participants’ height. Some studies have shown similar results^25–27^, whereas others have shown no age-related differences^28–30^. Leighton *et al*. showed that ocular AL growth stopped in young adulthood^31^, and Grosvenor *et al*. indicated that AL was shortened during aging^32^. The reason for AL shortening among older adults has not yet been clarified; however, Grosvenor and colleagues reported that it could occur due to the increasing refractive power caused by lens adjustment as a compensating normalisation mechanism^32^.

Notably, Ooi *et al*. reported no significant age-related difference in AL, and stated that shortening of the AL occurs due to compensation for the sharpness of the lens^33^. In this study, we report widespread ICT use compared with past studies^1^; therefore, the birth cohort effect should be considered, such that the overall environment for exposure to near work differs depending on age. Because this study was a cross-sectional survey, we were unable to determine whether the results were solely due to age differences from the birth cohort effect, or actual changes in biometry of the eye. Therefore, further prospective studies involving long-term follow-up are necessary.

The results of this study suggest that ocular AL might increase with ICT use. Interestingly, in this study, ocular AL was strongly related to ICT use for work, while private use was only weakly related. Notably, there was no significant relationship between game-playing and AL. Several studies have shown that game playing leads to improvements, rather than detrimental effects, on visual function^34,35–40^. Li *et al*. reported improvements in amblyopia in a study of 10 amblyopic adults who played video games^41^. The authors have suggested that playing a video game can normalise higher brain cortical function and correct any distorted retinal topography, which would improve visual information extraction abilities^42^. However, the smartphone is often placed closer to the eye than a traditional console video game; therefore, smartphone use may have an adverse effect on the eye. In this study, we did not separate time spent game-playing between video and smartphone games. Regarding private use, time spent watching TV could have affected the results. However, the TV presumably differs from the ICT for the purpose of this evaluation because of the large distance between the TV and the eyes, as well as the lack of staring during viewing, relative to other information terminals.

In addition, we confirmed the relationship between differences in left and right ocular AL and ICT use. Furthermore, elongation of ocular AL was significantly related to the time reported for word processing and email tasks. This result suggests that work involving long periods of staring at a screen with either dominant eye might lead to elongation of ocular AL, resulting in a lateral difference. In contrast, game playing may require binocular vision, and was not associated with left-right differences or elongation of AL. Based on these findings, we speculate that older participants required longer periods of staring at a screen with a dominant eye; this may be related to elongation of the AL, and the relationship with the dominant eye should be considered in a future study.

The detailed mechanisms by which ocular AL is extended due to ICT usage are unknown. It is possible that, during near-vision work, the nearness causes the eye to experience hyperopic defocus. Although the accommodation system reduces this hyperopic defocus, it is not entirely eliminated^43^. Stimulation of the retina with a blurred image results in alteration of the growth signals within the eye. Rada *et al*. reported that the retina provides remodelling signals to the sclera, such that the eye alters its shape to place an image on the retina via emmetropisation^44^. Typically, this change is reversed when near-vision work is completed. However, when exposed to chronic near-vision work, such as ICT use for an extended period, defocus information is aggregated across the entire surface of the retina and the integrated signal alters the growth of the eye; thus, ocular AL may increase^5^.

Furthermore, we found that the risk of glaucoma significantly increased with greater ocular AL. In this study, information regarding history of glaucoma was obtained by self-reporting, which may constitute a limitation. However, the participants in this study underwent annual glaucoma screening with frequency doubling technology perimetry and fundus examinations^22,24^. Thus, we presume that accurate information was included regarding glaucoma. Based on these considerations, our findings are consistent with previous reports that axial myopia is a risk factor for glaucoma^11–18^. In addition, it has been reported that tension is generated by stretching the AL, and that blood flow disorders around the optic nerve may be involved^19–21^; however, detailed mechanisms have not yet been elucidated.

The strength of this study is that it is the first to perform a detailed examination of ICT use in a large-scale population-based context, targeting workers who perform ICT work for both business and private purposes. Notably, the study included separate analyses for each purpose.

However, there were some limitations in this study. First, the study used a cross-sectional observation protocol. Here, AL obviously differed depending on age, and the birth cohort effect may have affected the findings. Therefore, in order to more accurately evaluate the influence of ICT use, a longitudinal cohort is needed, for which the same clinician measures AL over time. Second, the study may have excluded the influence of some factors on the results, such as family history of myopia, history of close work (e.g., reading), watching TV, outdoor physical activity, and age at which ICT devices were first used. Furthermore, in the analysis of ICT use for work purposes, it is difficult to exclude mutual entanglement because the majority of participants performed multiple types of work. Furthermore, the survey focused on ICT use at a single point in time; thus, it cannot evaluate changes in ICT use relative to past habits. An investigation of the history of detailed ICT use is needed in a future cohort study, in order to accurately assess past and present exposure. Third, this study only included detailed analysis of data from men, because there were limited data available regarding women. We cannot completely deny the possibility of a sex difference. Although stratified analysis by age could not be performed, among participants of all ages, a significant association of AL elongation with ICT use was observed (Supplemental Table 3). Thus, we presume that results obtained from analyses of men can be generalised to women.

Despite these limitations, our study clearly demonstrated that extended ICT use was related to elongation of ocular AL. This result is in agreement with our past findings regarding ICT use and the risk of glaucoma. Based on this result, we further hypothesise that elongation of ocular AL through long-term ICT use may be a risk factor for glaucoma. Therefore, longitudinal changes in ocular AL should be traced, in order to clarify the mechanism by which long-term ICT use leads to the onset of glaucoma.

## Methods

### Participants

This study was conducted at the Hitachi Health Care Center, which belongs to a group of large-scale electronics companies. Employees (“workers”) and their spouses (≥35 to 65 years of age) from 35 affiliated companies (38,000 workers) freely selected the timing and health centres where they would undergo comprehensive health examinations, including cardiovascular and cancer screening. Most workers were employed by electronics-related companies and performed generally representative (not industry-specific) jobs; these included general affairs, design, research and development, sales, and manufacturing.

Information regarding AL length was obtained as part of the following project: “Development for a glaucoma mass screening program using the Optical Coherence Tomography (OCT)”^45^.

Of 14573 workers who underwent health examinations between December 18, 2015 and February 15, 2017, 8989 were arbitrarily (according to reception order) recruited for this study because of time constraints. All 8989 workers (7661 men and 1328 women) provided informed consent to participate in our study.

Of the 8989 workers, 383 were excluded from the analysis: 312 lacked AL data in at least one eye, 43 exhibited AL of <20 mm in at least one eye, and 28 did not answer all inquiries in the questionnaire used in the study. Participants with AL of <20 mm were excluded because this length may cause the AL assessment device to show a signal in front of the retina (i.e., in the vitreous body or the high vitreous detachment). Finally, 8606 participants remained; of these, we performed detailed analyses of 7334 men, because there were a limited number of female employees to analyse. For reference, 1272 women were analysed.

This study was reviewed and approved in advance by the Hitachi Hospital Group Ethics Committee (Approval No. 2014-63, December 19, 2014) and Tokai University Ethics Committee (Approval No. 14-519, January 23, 2015). The described research adhered to the tenets of the Declaration of Helsinki.

### Axial length measurement

In accordance with the manufacturer’s instructions, the AL of both eyes was measured by using an optical axial length meter (Aladdin, Topcon, Tokyo, Japan) by the same trained operator for all participants in this study. Aladdin meters were provided by Topcon Co., Ltd.

### ICT use and ophthalmological questionnaire

Information regarding ICT use was obtained using a self-administered questionnaire that assessed the average number of hours of ICT use per day, while working and outside of work (private use), for the past 5 years. The number of hours was divided into the following five categories: <1 hour, 1–4 hours, 4–8 hours, 8–12 hours, and >12 hours. In addition, we assessed ICT use for each type of task (computer-aided design and computer-aided manufacturing, programming, operator job, word processing, designer, assembling presentation materials, sending e-mails, and browsing websites). For private use, the tasks included playing a game, sending e-mail, and browsing web sites. Additionally, we assessed history of work and private use of ICT using the following four categories: <5 years, 5–10 years, 10–20 years, and >20 years. Finally, we acquired information regarding history of glaucoma and ocular hypertension, and family history of glaucoma, using the self-administered questionnaire.

### Physical activity

In accordance with our previous report^46^, information on physical activity was obtained from the questionnaire (for example, whether participants regularly exercised during leisure, as well as the frequency of exercise [times per month, up to three activities] and time per day [hours]). When participants’ preferred activities were not listed, activities of similar strengths could be selected. Of the 20 types of exercise listed, “other” was not used in this analysis. Metabolic equivalents (METs) of each activity, based on physical activity guidelines, were assigned (if the MET value was not on the list, a MET value from a related exercise was used)^47^. Of the 19 exercises, 13 (work and commuting, walking, swimming, golf practice, golf, baseball, softball, bicycle cycling, table tennis, bread, badminton, strength training, light jogging [approximately 6 minutes/km], jogging, soccer, tennis, aerobics, and jump rope) were categorised as active activities (>6 METs); for these activities, weekly and hourly METs were calculated. Occupational physical activity was evaluated from among four options (mainly sitting, mainly standing, frequently walking, or active), and walk time for commuting was self-reported.

### Statistical analysis

For the background characteristics, continuous variables were described using mean ± standard deviation (SD). Among ICT use groups, statistical significance was determined by one-way ANOVA and Bonferroni comparisons. Categorical data on ICT use, history of intraocular pressure (IOP), and family history of glaucoma were examined by chi-squared test. To determine confounding factors, Pearson correlation analyses for parametric variables or Spearman correlation analyses for non-parametric variables were performed using all variables.

In this study, we analysed the right eye AL using ANCOVA and logistic analysis. Data in this study suggested that AL could be associated with age, participant’s height, and history of high IOP. Because physical activity was previously reported to be associated with AL^8^, physical activity was included in the analysis. First, to assess the association between AL and ICT use, we conducted ANCOVA with height as a covariate. In addition, to evaluate birth cohort effect, participants were separated using 5-year age intervals. We confirmed that there was no interaction between ICT use and height before running the full model.

To adjust for confounding factors, multiple logistic regression analysis was applied to examine the association between AL and ICT use. The high AL group was determined as above the 75th percentile for each 5-year age interval. In model 1, age and height were used as independent variables; in model 2, independent variables comprised age, height, physical activity, and history of high IOP. Odds ratios (ORs) were calculated for computer-aided design & computer-aided manufacturing, programming, operator job, word processing, designer, assembling presentation materials, sending e-mails, and browsing websites. Three (<1 hour, 1–4 hours, and <4 hours) or four categories (<1 hour, 1–4 hours, 4–8 hours, and <8 hours) were used because the numbers of the groups with 4–8 hours and >12 hours were small.

The difference in AL between the right and left eyes was examined by calculating the absolute value of right minus left AL. Significance was determined using ANOVA with 3 ICT use categories (<1 hour, 1–4 hours, and >4 hours) and Bonferroni correction. To investigate the association between AL and glaucoma, multiple logistic analyses were performed using age, height, history of IOP, family history of glaucoma, and AL as variables. AL was included in the model as a categorical variable based on 25th percentiles. All statistical analyses were performed using IBP-SPSS ver. 24 (IBM, Tokyo, Japan). Data were considered statistically significant when P < 0.05.

## Supplementary information

supplemental table

## Data Availability

The data are not available for public access because of patient privacy concerns, but are available from the corresponding author on reasonable request.
