# Supplementary material for: Association between Information and Communication Technology use and Ocular Axial Length Elongation among Middle-Aged Male Workers
Source: Sci Rep. 2019 Nov 25;9:17489. doi: 10.1038/s41598-019-53423-8 (PMC6877562; doi:10.1038/s41598-019-53423-8)
Supplement: Supplementary file 1 — supplemental table [file 41598_2019_53423_MOESM1_ESM.docx]

**Association between Information and Communication Technology use and Ocular Axial Length Elongation among Middle-Aged Male Workers**

Authors: Toru Honda^1)3)^, Toru Nakagawa^1)^, Yuya Watanabe^1)^, Takeshi Hayashi^1)^, Tadashi Nakano^2)^, Seichi Horie^3)^ and Masayuki Tatemichi^3),4)*^

1) Hitachi Health Care Center, Ibaraki, Japan

2) Department of Ophthalmology, The Jikei University School of Medicine, Tokyo, Japan

3) Department of Health Policy and Management, University of Occupational and Environmental Health, Kitakyushu, Japan

4) Department of Preventive Medicine, Tokai University, School of Medicine, Kanagawa, Japan

***Corresponding author:** Masayuki Tatemichi M.D., Ph. D.

Department of Preventive Medicine, Tokai University School of Medicine

143 Shimokasuya, Isehara, Kanagawa 259-1193, Japan

**Tel:** +81-463-93-1121 ex2621

**Fax:** +81-463-92-3549

**E-mail:** tatemichi@tokai-u.jp

| Supplement Table 1. Characteristics of Participants | | | | | | | | | | |
| --- | --- | --- | --- | --- | --- | --- | --- | --- | --- | --- |
|  |  |  |  |  |  | Age |  |  |  |  |
|  |  |  | 35-40 y | 41-45 y | 46-50 y | 51-55y | 56-60y | 61-65 y | *p* | Total |
| Number |  |  | 834 | 1498 | 1567 | 1356 | 1107 | 972 |  | 7334 |
| Age |  | mean | 37.7 | 43.2 | 47.9 | 53.0 | 57.8 | 63.1 | <0.001* | 50.2 |
|  |  | SD | 1.8 | 1.3 | 1.4 | 1.4 | 1.4 | 1.5 |  | 7.9 |
| Hight (cm) |  | mean | 172.7 | 172.4 | 172.0 | 171.0 | 169.9 | 168.2 | <0.001* | 171.2 |
|  |  | SD | 5.9 | 6.0 | 5.9 | 5.8 | 5.7 | 5.9 |  | 6.1 |
| Total ICT use time |  |  |  |  |  |  |  |  |  |  |
| per day |  | none or <1 hr. | 67 (8.0%) | 201 (13.4%) | 192 (12.3%) | 163 (12.0%) | 178 (16.1%) | 335 (34.5%) |  | 1136 (15.5%) |
|  |  | 1-4 hr. | 331 (39.7%) | 513 (34.2%) | 395 (25.2%) | 284 (20.9%) | 217 (19.6%) | 256 (26.3%) |  | 1996 (27.2%) |
|  |  | 4-8 hr. | 147 (17.6%) | 286 (19.1%) | 427 (27.2%) | 445 (32.8%) | 384 (34.7%) | 281 (28.9%) |  | 1970 (26.9%) |
|  |  | ≧8 hr. | 289 (34.7%) | 498 (33.2%) | 553 (35.3%) | 464 (34.2%) | 328 (29.6%%) | 100 (10.3%) | <0.001** | 2232 (30.4%) |
|  |  |  | 100% | 100% | 100% | 100% | 100% | 100% |  | 100% |
| Axial length (right) (mm) |  | mean | 25.29 | 25.16 | 25.20 | 25.13 | 25.08 | 24.74 | <0.001* | 25.11 |
|  |  | SD | 1.40 | 1.43 | 1.44 | 1.54 | 1.47 | 1.44 |  | 1.47 |
| Axial length (left) (mm) |  | mean | 25.24 | 25.11 | 25.14 | 25.06 | 25.03 | 24.67 | <0.001* | 25.05 |
|  |  | SD | 1.37 | 1.40 | 1.43 | 1.53 | 1.45 | 1.43 |  | 1.45 |
| *Statistical significance was determined by one-way ANOVA | | | | | | | | | | |
| ** Statistical significance was determined by Chi-squired test | | | | | | | | | | |
| SD=standard deviation | | | | | | | | | | |

| Supplement Table 2. Association between work related ICT use time and axial length elongation by stratification with age | | | | | | | | | | | | | | | | | | | |
| --- | --- | --- | --- | --- | --- | --- | --- | --- | --- | --- | --- | --- | --- | --- | --- | --- | --- | --- | --- |
|  | ICT Use Time/day |  |  |  | CAD/CADAM | |  |  |  |  | Programming | |  |  |  |  | Operator job | |  |
| Age |  |  | n | Odds | 95% CI | | *p* |  | n | Odds | 95% CI | | *p* |  | n | Odds | 95% CI | | *p* |
| 35-40 y | none or <1 hr. |  | 619 |  | Reference | |  |  | 706 |  | Reference | |  |  | 652 |  | Reference | |  |
|  | 1-4 hr. |  | 143 | 1.06 | 0.70 | 1.62 | 0.781 |  | 81 | 1.02 | 0.60 | 1.75 | 0.929 |  | 120 | 0.77 | 0.47 | 1.24 | 0.273 |
|  | ≧4 hr. |  | 67 | 1.06 | 0.59 | 1.91 | 0.836 |  | 42 | 1.30 | 0.65 | 2.61 | 0.458 |  | 57 | 1.45 | 0.80 | 2.62 | 0.217 |
|  |  |  |  | *p for trend =0.761* | | | |  |  | *p for trend =0.515* | | | |  |  | *p for trend =0.659* | | | |
| 41-45 y | none or <1 hr. |  | 1152 |  | Reference | |  |  | 1323 |  | Reference | |  |  | 1181 |  | Reference | |  |
|  | 1-4 hr. |  | 207 | 0.97 | 0.68 | 1.38 | 0.857 |  | 113 | 0.98 | 0.62 | 1.54 | 0.936 |  | 208 | 0.89 | 0.63 | 1.26 | 0.510 |
|  | ≧4 hr. |  | 134 | 1.54 | 1.05 | 2.26 | 0.029 |  | 57 | 1.32 | 0.73 | 2.36 | 0.358 |  | 104 | 0.90 | 0.56 | 1.44 | 0.652 |
|  |  |  |  | *p for trend=0.074* | | | |  |  | *p for trend =0.479* | | | |  |  | *p for trend =0.486* | | | |
| 46-50 y | none or <1 hr. |  | 1198 |  | Reference | |  |  | 1337 |  | Reference | |  |  | 1169 |  | Reference | |  |
|  | 1-4 hr. |  | 188 | 0.77 | 0.53 | 1.12 | 0.173 |  | 134 | 0.97 | 0.64 | 1.47 | 0.887 |  | 275 | 0.87 | 0.64 | 1.20 | 0.401 |
|  | ≧4 hr. |  | 171 | 1.24 | 0.87 | 1.76 | 0.244 |  | 86 | 1.31 | 0.81 | 2.12 | 0.264 |  | 113 | 1.55 | 1.03 | 2.35 | 0.037 |
|  |  |  |  | *p for trend=0.592* | | | |  |  | *p for trend =0.379* | | | |  |  | *p for trend =0.220* | | | |
| 51-55 y | none or <1 hr. |  | 1032 |  | Reference | |  |  | 1151 |  | Reference | |  |  | 983 |  | Reference | |  |
|  | 1-4 hr. |  | 160 | 0.66 | 0.43 | 1.02 | 0.061 |  | 128 | 0.60 | 0.37 | 0.98 | 0.041 |  | 247 | 0.84 | 0.60 | 1.17 | 0.301 |
|  | ≧4 hr. |  | 152 | 1.28 | 0.88 | 1.86 | 0.204 |  | 65 | 1.06 | 0.60 | 1.88 | 0.834 |  | 114 | 1.09 | 0.70 | 1.70 | 0.695 |
|  |  |  |  | *p for trend=0.637* | | | |  |  | *p for trend =0.368* | | | |  |  | *p for trend =0.515* | | | |
| 56-60 y | none or <1 hr. |  | 821 |  | Reference | |  |  | 962 |  | Reference | |  |  | 820 |  | Reference | |  |
|  | 1-4 hr. |  | 116 | 0.78 | 0.48 | 1.27 | 0.327 |  | 81 | 0.76 | 0.43 | 1.34 | 0.335 |  | 162 | 1.04 | 0.70 | 1.53 | 0.856 |
|  | ≧4 hr. |  | 151 | 1.51 | 1.03 | 2.20 | 0.033 |  | 45 | 1.48 | 0.78 | 2.81 | 0.231 |  | 106 | 1.34 | 0.86 | 2.09 | 0.203 |
|  |  |  |  | *p for trend=0.094* | | | |  |  | *p for trend =0.610* | | | |  |  | *p for trend =0.849* | | | |
| 61-65 y | none or <1 hr. |  | 574 |  | Reference | |  |  | 660 |  | Reference | |  |  | 581 |  | Reference | |  |
|  | 1-4 hr. |  | 104 | 0.66 | 0.39 | 1.11 | 0.115 |  | 67 | 0.98 | 0.55 | 1.77 | 0.959 |  | 108 | 0.98 | 0.61 | 1.58 | 0.927 |
|  | ≧4 hr. |  | 83 | 1.30 | 0.79 | 2.13 | 0.296 |  | 34 | 1.35 | 0.64 | 2.84 | 0.425 |  | 72 | 2.21 | 1.34 | 3.65 | 0.002 |
|  |  |  |  | *p for trend =0.780* | | | |  |  | *p for trend=0.528* | | | |  |  | *p for trend =0.247* | | | |
|  | | | | | | | | | | | | | | | | | | | |
|  |  |  |  |  |  |  |  |  |  |  |  |  |  |  |  |  |  |  |  |

(Continued)

| Supplement Table 2. Association between work related ICT use time and axial length elongation by stratification with age | | | | | | | | | | | | | |
| --- | --- | --- | --- | --- | --- | --- | --- | --- | --- | --- | --- | --- | --- |
|  | ICT Use Time/day |  |  | Designer | | | |  | Browsing websites | | | | |
| Age |  |  | n | Odds | 95% CI | | *p* |  | n | Odds | 95% CI | | *p* |
| 35-40 y | none or <1 hr. |  | 747 |  | Reference | |  |  | 363 |  | Reference | |  |
|  | 1-4 hr. |  | 64 | 1.19 | 0.67 | 2.12 | 0.547 |  | 444 | 1.78 | 1.27 | 2.49 | 0.001 |
|  | ≧4 hr. |  | 18 | 0.89 | 0.29 | 2.74 | 0.836 |  | 22 | 1.62 | 0.61 | 4.29 | 0.336 |
|  |  |  |  | *p for trend=0.804* | | | |  |  | *p for trend=0.001* | | | |
| 41-45 y | none or <1 hr. |  | 1384 |  | Reference | |  |  | 672 |  | Reference | |  |
|  | 1-4 hr. |  | 86 | 1.84 | 1.16 | 2.92 | 0.010 |  | 783 | 2.22 | 1.72 | 2.85 | <0.001 |
|  | ≧4 hr. |  | 23 | 2.45 | 1.05 | 5.70 | 0.038 |  | 38 | 1.03 | 0.44 | 2.42 | 0.938 |
|  |  |  |  | *p for trend=0.001* | | | |  |  | *p for trend <0.001* | | | |
| 46-50 y | none or <1 hr. |  | 1433 |  | Reference | |  |  | 545 |  | Reference | |  |
|  | 1-4 hr. |  | 101 | 1.15 | 0.72 | 1.81 | 0.559 |  | 963 | 1.85 | 1.43 | 2.41 | 0.000 |
|  | ≧4 hr. |  | 23 | 1.34 | 0.55 | 3.29 | 0.525 |  | 49 | 2.24 | 1.19 | 4.25 | 0.013 |
|  |  |  |  | *p for trend=0.397* | | | |  |  | *p for trend <0.001* | | | |
| 51-55 y | none or <1 hr. |  | 1246 |  | Reference | |  |  | 376 |  | Reference | |  |
|  | 1-4 hr. |  | 73 | 1.21 | 0.71 | 2.06 | 0.481 |  | 920 | 1.61 | 1.20 | 2.17 | 0.002 |
|  | ≧4 hr. |  | 25 | 1.31 | 0.54 | 3.18 | 0.551 |  | 48 | 1.18 | 0.56 | 2.48 | 0.663 |
|  |  |  |  | *p for trend=0.371* | | | |  |  | *p for trend =0.011* | | | |
| 56-60 y | none or <1 hr. |  | 995 |  | Reference | |  |  | 307 |  | Reference | |  |
|  | 1-4 hr. |  | 71 | 1.19 | 0.69 | 2.05 | 0.523 |  | 720 | 1.97 | 1.41 | 2.76 | <0.001 |
|  | ≧4 hr. |  | 22 | 2.26 | 0.95 | 5.39 | 0.065 |  | 61 | 2.32 | 1.25 | 4.31 | 0.008 |
|  |  |  |  | *p for trend=0.074* | | | |  |  | *p for trend <0.001* | | | |
| 61-65 y | none or <1 hr. |  | 708 |  | Reference | |  |  | 290 |  | Reference | |  |
|  | 1-4 hr. |  | 36 | 0.88 | 0.40 | 1.97 | 0.762 |  | 446 | 1.73 | 1.27 | 2.34 | <0.001 |
|  | ≧4 hr. |  | 17 | 1.91 | 0.71 | 5.12 | 0.197 |  | 25 | 1.89 | 0.79 | 4.53 | 0.152 |
|  |  |  |  | *p for trend=0.037* | | | |  |  | *p for trend <0.001* | | | |
| The high AL group was determined as above the 75th percentile for each 5-year age interval. The odds ratio for the high AL group was calculated using the logistic model after adjusted for age and height. 95%CI=95% confidence interval | | | | | | | | | | | | | |
|  |  |  |  |  |  |  |  |  |  |  |  |  |  |

| Supplement Table 3. Association between ICT use time and axial length elongation among women | | | | | | | | |  |  |
| --- | --- | --- | --- | --- | --- | --- | --- | --- | --- | --- |
| ICT Use Time/day |  |  | model 1 | |  |  | model 2 | | | |
|  | n | Odds | 95% CI | | p |  | Odds | 95% CI | | p |
| none or <1 hr. | 365 |  | Reference | |  |  |  | Reference | |  |
| 1-4 hr. | 359 | 1.05 | 0.71 | 1.54 | 0.817 |  | 1.05 | 0.72 | 1.55 | 0.789 |
| 4-8 hr. | 154 | 0.97 | 0.60 | 1.57 | 0.899 |  | 0.95 | 0.58 | 1.54 | 0.819 |
| ≧ 8hr | 145 | 2.01 | 1.26 | 3.20 | 0.003 |  | 1.95 | 1.21 | 3.12 | 0.006 |
|  |  | *p* for trend　<0.012 | | | |  | *p* for trend　<0.021 | | | |
| The high AL group was determined as above the 75th percentile for each 5-year age interval. The odds ratio for the high AL group was calculated using the logistic model | | | | | | | | | | |
|  |  |  |  |  |  |  |  |  |  |  |
| Model 1：Abjuseted for age, height | | |  |  |  |  |  |  |  |  |
| Model 2: Adjusted for age, height, exercise, and history of ocular hypertension | | | | | | | |  |  |  |
| 95%CI=95% confidence interval | | |  |  |  |  |  |  |  |  |
